# Prevalence of accessory navicular in Japanese children: A cross-sectional study using ultrasound–Katsuragi Integrated Defense for Locomotive Syndrome in children study

**DOI:** 10.1371/journal.pone.0318014

**Published:** 2025-01-24

**Authors:** Takahide Sasaki, Masatoshi Teraguchi, Kanae Mure, Yoshiki Asai, Yusuke Kido, Makiko Onishi, Takashi Shimoe, Nobuyuki Miyai, Yukihiro Nakagawa, Hiroshi Hashizume, Hiroshi Yamada

**Affiliations:** 1 Department of Orthopaedic Surgery, Kihoku Hospital, Wakayama Medical University, Katsuragi Town, Wakayama, Japan; 2 Department of Orthopaedic Surgery, Wakayama Medical University, Wakayama City, Wakayama, Japan; 3 Department of Public Health, Wakayama Medical University, Wakayama City, Wakayama, Japan; 4 Department of Orthopedic Surgery, Aitoku Medical and Welfare Center, Wakayama City, Wakayama, Japan; 5 Faculty of Health and Nursing, Wakayama Medical University, Wakayama City, Wakayama, Japan; Cairo University Faculty of Physical Therapy, EGYPT

## Abstract

The accessory navicular (AN) is an accessory bone located on the posteromedial aspect of the navicular tuberosity that can cause pain following overuse or trauma, particularly during childhood. However, the detailed epidemiological characteristics of AN in children have not been well studied. This study aimed to clarify the prevalence of AN and painful AN among Japanese children by examining the characteristics according to sex and age. This cross-sectional study used data from the Katsuragi Integrated Defense for Locomotive Syndrome in Children Study, focusing on musculoskeletal disorders in 875 children aged 6–15 years, with 1750 feet being assessed. Children were divided into five age groups: 6–7, 8–9, 10–11, 12–13, and 14–15. AN was detected using ultrasound to avoid radiation exposure. The sex- and age-group-dependent prevalence of AN and painful AN were calculated, and statistical analyses examined sex differences in prevalence by age group. The overall prevalence of AN was 15.1%, higher in females (17.9%) than in males (12.3%). The prevalence of AN increased with age in both sexes. Among cases diagnosed with AN, 20.8% were symptomatic, with a unimodal peak observed at ages 12–13 in males and 10–11 in females. No statistically significant differences were observed in the proportion of painful AN between sexes. This is the first large-scale epidemiological study on AN in children. The overall prevalence of AN was 15.1%, higher in females than in males. Additionally, 20.8% of patients with AN experienced pain. The results of this study provide important epidemiological data to support clinical management strategies for pediatric patients with AN.

## Introduction

The accessory navicular (AN) is an accessory bone located on the posteromedial aspect of the navicular tuberosity and is considered a developmental variation of the secondary ossification center [1]. The AN can cause foot pain following overuse or trauma [2], typically on the medial side of the foot during puberty [1]. Conservative treatments for managing painful AN include observation, physical therapy, oral nonsteroidal pain medication, and orthotics [2–4]. In these failures, surgical treatments such as simple excision of AN, percutaneous drilling, or the Kidner procedure are indicated [5–8].

AN is a commonly identified ossicle in the foot [7,9], with an incidence ranging from 4% to 21% [5,9–11]. Although often symptomatic during childhood [4], few epidemiological studies have focused on its prevalence and associated pain in this age group. Most epidemiological research has been conducted on adults [2,9,10], and existing studies on children have limitations such as small sample sizes and selection bias due to the use of patient-based samples [2,12]. Thus, the detailed epidemiological characteristics of AN in children remain unclear, necessitating further research [2,12]. To the best of our knowledge, no large-scale epidemiological studies have investigated AN in the general pediatric population.

This study aimed to conduct a comprehensive epidemiological survey of Japanese children to identify epidemiological indicators of AN. Specifically, it sought to determine: (1) the prevalence of AN in Japanese children and its characteristics by sex and age, (2) the proportion of painful AN and its characteristics by sex and age. The results could provide fundamental information to assist clinicians in determining treatment strategies for patients with painful AN.

## Materials and methods

### Study design

This cross-sectional study investigated the epidemiological indicators of AN in a Japanese population of children. This study complied with the principles of the Declaration of Helsinki and was approved by the Ethics Committee of Wakayama Medical University (No.3594). Written informed consent was obtained from the parents or guardians of all participants.

### Participants

The Katsuragi Integrated Defense for Locomotive Syndrome in Children Study (KID Locomo Study) is a prospective cohort study aimed at elucidating musculoskeletal disorders in childhood, identifying risk factors, and establishing preventive methods. The study participants included children from five elementary schools and two middle schools in Katsuragi Town, Wakayama Prefecture. A baseline survey was conducted in each elementary and middle school between October 2022 and February 2023. One small elementary school was surveyed together with the other schools because of its small student population. Annual follow-up surveys were conducted. A team of orthopedic surgeons, nurses, physical therapists, university faculty, and graduate students collected the data. Participants received a data collection manual and attended rehearsal sessions to standardize the protocol.

This cross-sectional analysis of the second KID Locomo Study was conducted from September 2023 to January 2024. Of the 1,037 children from elementary and junior high schools in Katsuragi Town, 875 (446 males and 429 females) participated in this study, excluding those who did not consent or were absent. The exclusion criteria were as follows: (1) medical diseases or musculoskeletal disorders that precluded physical function testing, (2) internal metal implants that precluded body composition testing, and (3) history of foot surgery or paralysis. No children met the exclusion criteria; therefore, the study included 875 children (446 males and 429 females) aged 6–15 years, encompassing 1,750 feet.

### Measurements

The KID Locomo Study collected data on: (1) height and weight, (2) physical function tests (two-step test, stand-up test, standing on one leg, squatting, arm raising, forward flexion), (3) body composition using bioelectrical impedance analysis (MC780-AN, Tanita Corp., Tokyo, Japan), (4) bone density of the calcaneus using quantitative ultrasound (Benus Ⅲ, Ishikawa Seisakusho, Kanazawa, Japan), (5) posture using a computer-assisted non-invasive device (Spinal Mouse, Idiag, Volkerswill, Switzerland), (6) fat degeneration of the quadriceps using ultrasound (a 11-MHz high-resolution linear-array transducer, SONIMAGE MX1, Konica Minolta, Tokyo, Japan), (7) presence of AN using ultrasound, (8) presence of flat foot based on footprint analysis (Sorbo Foot Printer, Sanshin Enterprises Co., Ltd., Tokyo, Japan), and (9) various information collected through a range of standardized self-reported questionnaires (demographic information, questionnaire about lifestyle habits, exercise habits including club activities, shoulder stiffness, and lower back pain, Strengths and Difficulties Questionnaire (SDQ) [13], brief self-administered diet history questionnaire (BDHQ)-15y [14]). Height and weight were measured using a fixed stadiometer and digital scale, respectively, to compute body mass index (BMI). For participants identified as having AN through ultrasound examination, an interview was conducted to assess the presence of pain in the AN (at rest, during walking, or during exercise). This study analyzed demographic information, height, weight, presence of AN using ultrasound, and presence of pain in AN.

### Ultrasound measurements of accessory navicular

**Fig 1:**
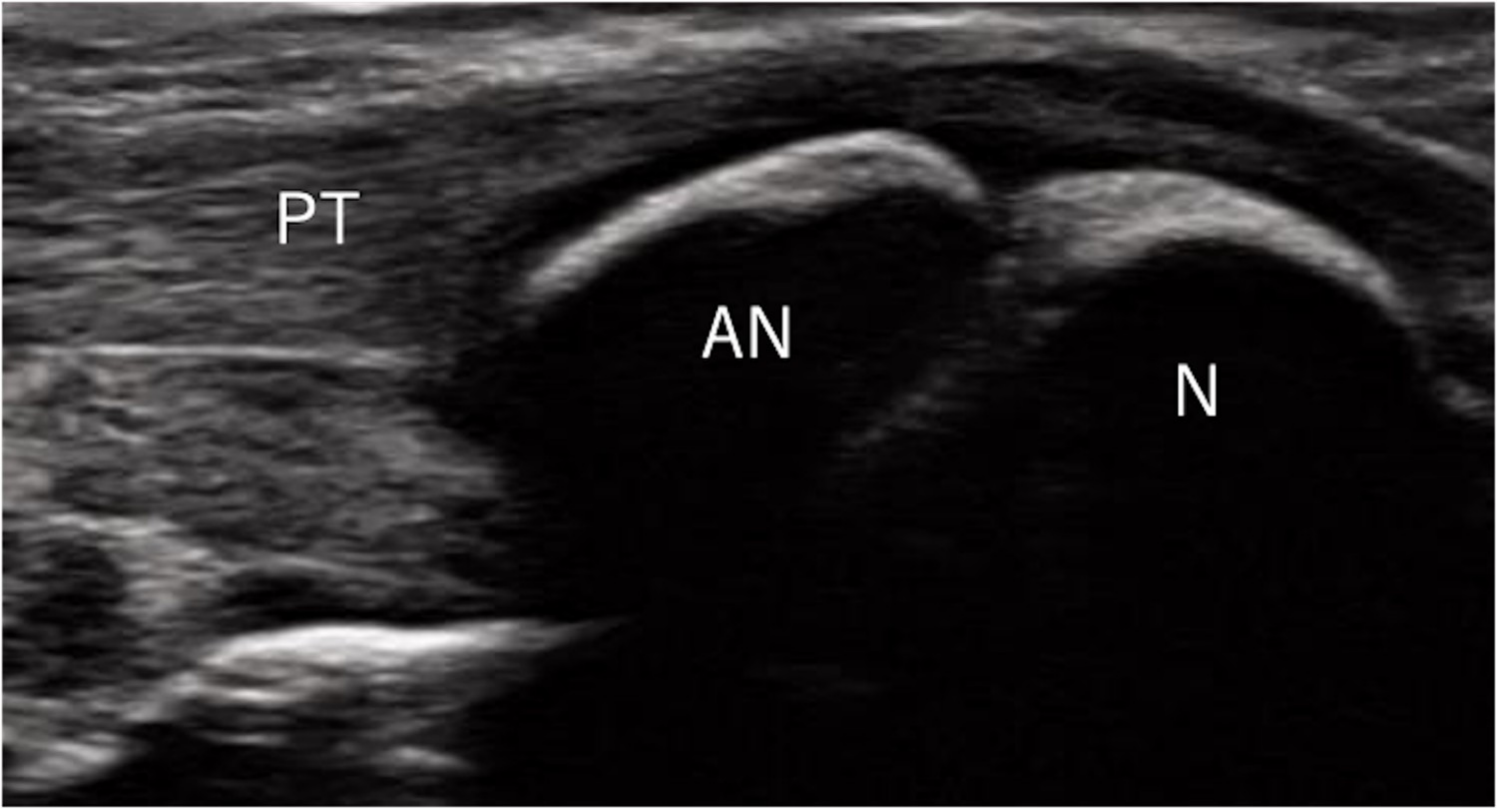
Ultrasonographic findings of the accessory navicular bone. The accessory navicular bone is identified on the proximal side of the navicular bone. Note: PT = posterior tibialis tendon; AN = accessory navicular; N = navicular.

As this study targeted a general population cohort of children, the presence of AN was assessed using ultrasound instead of X-rays to avoid radiation exposure. The evaluation of AN via ultrasound followed the methods reported in a previous study [15]. Participants were seated on a chair with slightly flexed hip and knee joints and feet in a frog-leg position, with the medial side facing up on the chair. The navicular tuberosity was first palpated, and the probe was placed directly over it along the foot’s longitudinal axis. The navicular tuberosity and posterior tibial tendon attachment were visualized, and the AN was depicted on the proximal medial side of the navicular (Fig 1). The navicular tuberosity was carefully examined from the plantar to the dorsal side for the presence of the AN. Additionally, because the type I AN is located within the posterior tibial tendon and is separated from the navicular region [1,7,16], the distal part of the posterior tibial tendon was meticulously observed for AN. Ultrasound examinations were performed on both feet.

The evaluation of AN via ultrasound was conducted at each elementary and junior high school by two board-certified foot and ankle surgeons (TS and YA), and the results were used for analysis. The ultrasound data were saved in video format, and 80 cases were randomly selected from the stored data. To calculate intraobserver and interobserver reliability, TS and YA independently evaluated each of the 80 cases, with all participant information blinded, separately from the initial on-site evaluations. TS performed assessments twice, with a 1-month interval between them, whereas YA performed a single assessment. Intra-observer reliability was calculated based on the two assessments conducted by TS. Interobserver reliability was calculated based on the first assessment by TS and the assessment by YA. A kappa value >0.90 was considered excellent, and that between 0.80 and 0.90 was considered good [17,18]. Disagreements in ultrasound image classification were resolved by consensus after reliability assessments were completed.

### Statistical analyses

Descriptive statistics summarized demographic characteristics, AN prevalence, and the proportion of painful AN. The prevalence of AN and the proportion of painful AN were calculated for the entire cohort and analyzed by sex and age groups. Age was classified into five categories: Group 1 (ages 6–7 years), Group 2 (ages 8–9 years), Group 3 (ages 10–11 years), Group 4 (ages 12–13 years), and Group 5 (ages 14–15 years). AN prevalence and the proportion of painful AN were calculated based on the number of feet. Chi-squared tests were used to compare the prevalence of AN and the proportion of painful ANs between sexes within the entire cohort and each age group. Among participants with AN, the proportion with bilateral ANs was calculated. All statistical analyses were performed using JMP version 14 (SAS Institute Japan, Tokyo, Japan). Statistical significance was set at p <0.05.

## Results

### Participant characteristics

Table 1 presents the demographic characteristics of the 875 participants, comprising 446 males (51.0%) with a mean age of 10.6 ± 2.6 years and 429 females (49.0%) with a mean age of 10.5 ± 2.6 years.

**Table 1:** Participant characteristics.

	Age (years)	6	7	8	9	10	11	12	13	14	15	Total
Male	Number of participants	16 (3.6)	48 (10.8)	49 (11.0)	47 (10.5)	63 (14.1)	42 (9.4)	51 (11.4)	48 (10.8)	60 (13.5)	22 (4.9)	446
	Demographic characteristics											
	•Height (cm)	120.9 ± 5.1	120.9 ± 5.9	130.6 ± 5.2	135.5 ± 6.1	139.9 ± 6.4	144.2 ± 8.6	150.2 ± 7.5	159.1 ± 8.6	164.9 ± 6.7	166.6 ± 3.0	144.0 ± 16.3
	•Weight (kg)	24.5 ± 4.7	24.6 ± 6.0	30.6 ± 7.3	32.1 ± 7.4	34.8 ± 7.3	38.1 ± 10.6	42.6 ± 8.0	48.9 ± 11.1	56.8 ± 10.9	53.6 ± 6.0	39.2 ± 13.5
	•Body mass index (kg/m^2^)	16.6 ± 2.0	16.7 ± 2.8	17.8 ± 3.3	17.4 ± 2.8	17.6 ± 2.7	18.1 ± 3.7	18.8 ± 2.7	19.2 ± 3.2	20.6 ± 3.5	19.3 ± 2.1	18.3 ± 3.3
Female	Number of participants	18 (4.7)	42 (9.8)	62 (14.5)	46 (10.7)	48 (11.2)	47 (11.0)	45 (10.5)	52 (12.1)	51 (11.9)	18 (4.2)	429
	Demographic characteristics											
	•Height (cm)	118.6 ± 4.9	120.9 ± 5.4	127.7 ± 6.5	133.6 ± 7.6	141.9 ± 7.6	150.6 ± 6.1	155.5 ± 6.4	156.5 ± 5.7	156.1 ± 5.3	157.0 ± 4.6	142.4 ± 15.1
	•Weight (kg)	22.7 ± 4.8	23.4 ± 4.8	26.5 ± 5.0	30.6 ± 6.5	35.9 ± 7.8	44.7 ± 11.2	47.6 ± 8.2	49.2 ± 11.8	48.9 ± 5.0	50.2 ± 10.0	38.1 ± 13.0
	•Body mass index (kg/m^2^)	16.1 ± 2.3	15.9 ± 2.4	16.1 ± 2.0	16.9 ± 2.3	17.7 ± 2.6	19.5 ± 3.8	19.6 ± 2.7	20.0 ± 4.1	20.1 ± 2.0	20.3 ± 3.6	18.2 ± 3.3

Data are presented as the mean ± standard deviation or as n (%).

### Sex- and age-strata-dependent prevalence of accessory navicular

**Fig 2:**
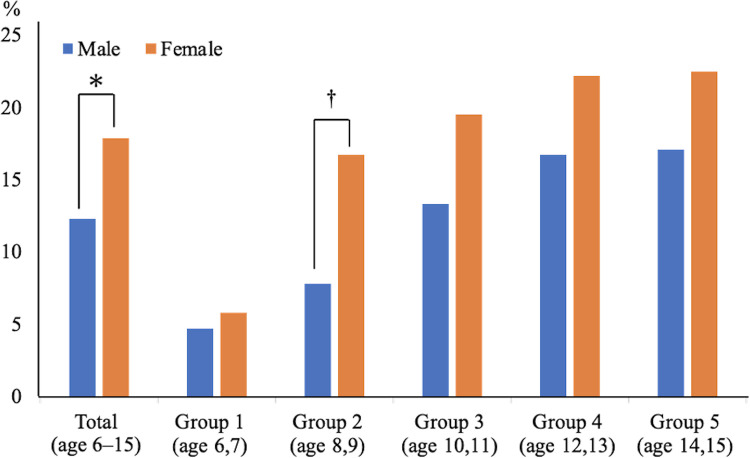
Sex- and age group-dependent prevalence of accessory navicular. * p = 0.001. † p <0.05.

Intraobserver and interobserver reliability were 0.94 and 0.91 for the ultrasound evaluation of AN, respectively. The prevalence of AN in the entire cohort was 15.1% (264 of 1,750 feet). The sex- and age-dependent prevalence of AN is shown in Fig 2. The overall prevalence of AN in the cohort was 12.3% (110 of 892 feet) in males and 17.9% (154 of 858 feet) in females, with females having a significantly higher prevalence than males (p = 0.001). The prevalence of AN in males was 4.7% in Group 1 (6 of 128 feet), 7.8% in Group 2 (15 of 192 feet), 13.3% in Group 3 (28 of 210 feet), 16.7% in Group 4 (33 of 198 feet), and 17.1% in Group 5 (28 of 164 feet). In females, the prevalence was 5.8% in Group 1 (7 of 120 feet), 16.7% in Group 2 (36 of 216 feet), 19.5% in Group 3 (37 of 190 feet), 22.2% in Group 4 (43 of 194 feet), and 22.5% in Group 5 (31 of 138 feet). In Group 2, females had a significantly higher prevalence than males (p <0.05). Although no statistically significant differences were observed in the other age groups, females consistently had a higher prevalence of AN across all age groups. The graph in Fig 2 shows a trend of increasing AN prevalence with increasing age in both males and females. Among participants with AN, 60% had bilateral AN (99 bilateral vs. 66 unilateral out of 165 total).

### Sex- and age-strata-dependent proportion of painful accessory navicular

**Fig 3:**
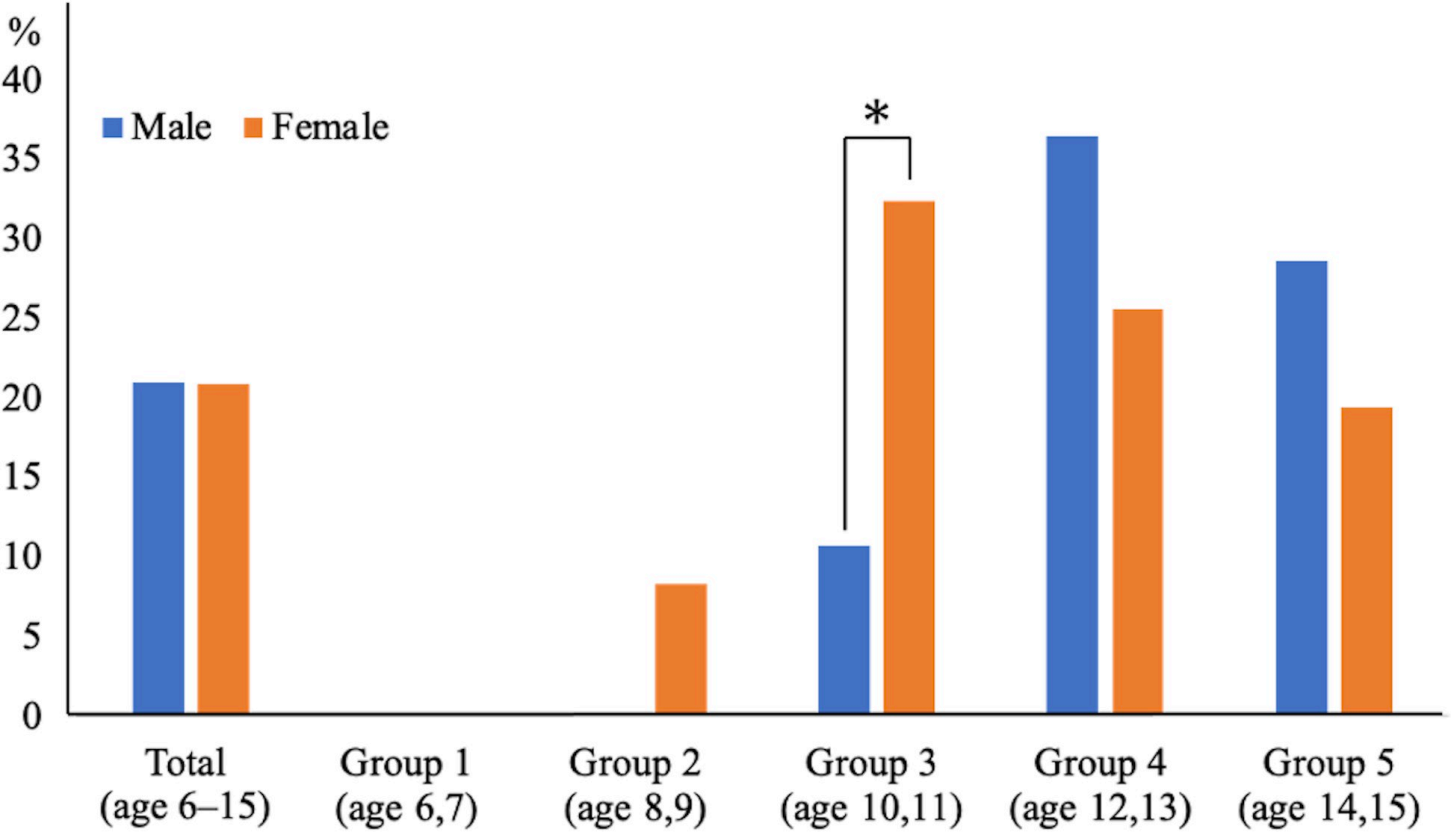
Sex- and age group-dependent proportion of painful accessory navicular. * p <0.05.

In the entire cohort, the proportion of painful AN was 20.8% (55 of 264 feet). This proportion was 20.9% in males (23 of 110 feet) and 20.8% in females (32 of 154 feet), showing no significant sex differences. The sex- and age-group-dependent proportions of painful AN are shown in Fig 3. The proportions of painful AN in males were as follows: 0% in Group 1 (0 of 6 feet), 0% in Group 2 (0 of 15 feet), 10.7% in Group 3 (3 of 28 feet), 36.4% in Group 4 (12 of 33 feet), and 28.6% in Group 5 (8 of 28 feet). In females, the proportions were 0% in Group 1 (0 of 7 feet), 8.3% in Group 2 (3 of 36 feet), 32.4% in Group 3 (12 of 37 feet), 25.6% in Group 4 (11 of 43 feet), and 19.4% in Group 5 (6 of 31 feet). In Group 3, females had a significantly higher proportion of painful AN than males (p <0.05). In the other groups, no significant sex differences were observed in the proportion of painful AN. The graph in Fig 3 shows a unimodal trend in the proportion of painful ANs, peaking in Group 4 (ages 12–13 years) for males and in Group 3 (ages 10–11 years) for females.

## Discussion

This study revealed that the prevalence of AN was 15.1% in a cohort of Japanese children, with a higher prevalence in females than in males. Additionally, the prevalence increased with age in both sexes. 20.8% of the AN cases were painful, with a unimodal peak observed at ages 12–13 in males and 10–11 in females. This is the first large-scale epidemiological investigation of AN in a general pediatric population, providing fundamental epidemiological indicators for AN prevalence in Japanese children. Furthermore, this study is the first to clarify the proportion of painful AN by sex and age in children. The results of this study could help clinicians treat patients with painful AN.

In this study, the prevalence of AN in the entire cohort was 15.1% (264 feet out of 1,750 feet). The prevalence of AN has previously been shown to vary from 4% to 21% [5,9–11], with the wide range attributed to differences in race and measurement methods [19]. Only one epidemiological study of AN has been conducted in the Japanese population. Tsuruta et al. examined plain X-rays of 3,460 patients (3,460 feet) and reported AN in 733 feet (21.2%) [20]. The prevalence rate in our study was lower than that reported by Tsuruta et al., potentially owing to differences in study populations. Tsuruta et al. examined patients ≥8 years old who attended their clinic for various reasons. Although the age distribution of their participants was not specified, it is likely that many adults were included. AN typically appears at an average of 11.4 ± 2.0 years [2]. Our study’s inclusion of participants aged 5–15 years may have resulted in a higher proportion of those who had not yet developed an AN, explaining the lower prevalence of AN in our study than that reported by Tsuruta et al., despite both studies being conducted in Japanese populations.

In the present study, the prevalence of AN was higher in females than in males across all age groups. Several studies have addressed sex differences in the prevalence of AN. Alsager et al. investigated 117 patients (194 feet) ≥18 years old at an orthopedic foot and ankle clinic in Saudi Arabia using plain X-rays and found the prevalence of AN to be 19.3%, with a significantly higher incidence in females than in males [21]. Lee et al. examined the presence of accessory bones and tarsal coalitions in the foot and ankle in a healthy, asymptomatic Korean population of 448 individuals (896 feet) aged 7–69 years; they identified AN in 34% of the participants, with a significantly higher frequency in females than in males [19]. The results of the present study align with these previous findings. Regardless of race, AN may be more common in females than in males. In this study, focusing on children may have influenced sex differences identified in the prevalence of AN. AN typically appears at an average age of 12.2 ± 2.2 years in males and 10.1 ± 0.7 years in females, indicating females develop AN approximately 2 years earlier than males [2]. This earlier onset in females may explain the higher prevalence of AN in females across all age groups in this study.

In the present study, the prevalence of AN increased with age in both males and females. No studies have investigated the age-specific prevalence rates of AN in childhood. Two factors may explain this age-related increase. First, the timing of AN appearance could be a contributing factor. Knapik et al. reported that AN appears at an average age of 11.4 ± 2.0 years [2]. Consequently, in younger age groups, AN may not yet have developed, leading to an observed increase in prevalence with advancing age. Second, the fusion rate of AN may play a role. According to Knapik et al., the fusion rate of AN is approximately 42%, suggesting that some AN do not fuse and remain present [2]. Therefore, the presence of unfused AN may contribute to the increase in prevalence with age.

In this study, using non-invasive ultrasound instead of X-rays allowed us to avoid radiation exposure and conduct an epidemiological survey of AN in the general pediatric population. No previous epidemiological studies have used ultrasound to investigate AN. In this study, AN was observed in 4.7% of males and 5.8% of females aged 5–7 years. Tsuruta et al. reported the minimum age of AN appearance as 9 years for males and 8 years for females in the Japanese population [20]. In our study, AN was detected at an earlier age than that in a previous study. This difference may be attributed to variations in the measurement methods. Although previous studies used X-rays to measure AN, our study used ultrasound examination. Ultrasound has superior detection capabilities for localized bone lesions compared to X-rays [22–24], enabling the detection of minute bone fragments that are undetectable on plain X-rays.

In the present study, among the participants with AN, the proportion of those with pain was 20.8%, with no sex differences. A unimodal trend was observed, with the prevalence peaking at ages 12–13 years in males and 10–11 years in females. This is the first study to investigate the prevalence of painful AN in children according to sex and age. Previous studies have focused on adults and patient groups. Kalbouneh et al. examined the radiographs of 1,240 feet of individuals with foot pain and found AN in 20.9% (259 of 1240 feet) and AN-related pain in 10.6% (26 of 246 feet) [25]. Knapik et al. reported symptoms in 0.1% of adult patients with AN [4]; although painful AN is rare in adults, this study found a higher proportion of painful AN in children than in adults. Additionally, the age-related trend in the proportion of painful AN was unimodal for both sexes, peaking earlier in females than in males. Inflammation and instability of synchondroses between the AN and navicular bone due to tension, shear, and/or compression forces cause painful AN [26,27]. Thirty-three percent of AN in males naturally fuse at an average age of 14.1 ± 2.7 years, whereas 57% of AN in females naturally fuse at an average age of 12.5 ± 1.0 years [2]. The disappearance of pain originating from the synchondrosis between the AN and navicular bone due to natural fusion might explain the observed trend in the proportion of painful AN in this study.

This study had some limitations. First, although it included >800 participants, they were recruited from only one region in Japan, which may not be representative of the general population. Second, due to its cross-sectional design, the study could not elucidate the natural history of AN or determine causal relationships between painful AN and anthropometric measurements. However, because the KID Locomo Study is a longitudinal survey, future work can help clarify these aspects. Third, the study population consisted of a single ethnic group, and thus, it is not possible to generalize the results to other ethnic groups. Fourth, AN was not classified using the Veitch classification, which has been reported to include many painful AN as Veitch Type II [7,28,29]. Fifth, pain variables, such as the Visual Analogue Scale and Numerical Rating Scale, were not evaluated in assessing pain associated with AN.

## Conclusion

This is the first large-scale, pediatric population-based study to examine the sex- and age-dependent prevalence of AN and the proportion of painful AN. The prevalence of AN was 15.1%, higher in females, and increased with age. The proportion of painful AN among AN cases was 20.8%, with no sex differences. A unimodal trend was observed, with prevalence peaking at ages 12–13 years in males and 10–11 years in females. The results provide fundamental epidemiological indicators of AN in children and crucial information for clinicians in determining treatment strategies for patients with painful AN. Further research incorporating this classification is expected to achieve a more detailed understanding of the characteristics and prevalence of painful AN.

## References

[1] Xiang F, Liu ZQ, Zhang XP, Li YJ, Wen J. Accessory navicular in children. World J Clin Cases. 2023;11(35):8256–62. doi: 10.12998/wjcc.v11.i35.8256 38130606 PMC10731211

[2] Knapik DM, Guraya SS, Conry KT, Cooperman DR, Liu RW. Longitudinal radiographic behavior of accessory navicular in pediatric patients. J Child Orthop. 2016;10(6):685–9. doi: 10.1007/s11832-016-0777-x 27807730 PMC5145827

[3] Fredrick LA, Beall DP, Ly JQ, Fish JR. The symptomatic accessory navicular bone: a report and discussion of the clinical presentation. Curr Probl Diagn Radiol. 2005;34(2):47–50. doi: 10.1067/j.cpradiol.2004.12.004 15753878

[4] Knapik DM, Archibald HD, Xie KK, Liu RW. A retrospective study on factors predictive of operative intervention in symptomatic accessory navicular. J Child Orthop. 2019;13(1):107–13. doi: 10.1302/1863-2548.13.180168 30838083 PMC6376442

[5] Wariach S, Karim K, Sarraj M, Gaber K, Singh A, Kishta W. Assessing the Outcomes Associated with Accessory Navicular Bone Surgery-a Systematic Review. Curr Rev Musculoskelet Med. 2022;15(5):377–84. doi: 10.1007/s12178-022-09772-5 35776339 PMC9463416

[6] Kopp FJ, Marcus RE. Clinical outcome of surgical treatment of the symptomatic accessory navicular. Foot Ankle Int. 2004;25(1):27–30. doi: 10.1177/107110070402500106 14768961

[7] Leonard ZC, Fortin PT. Adolescent accessory navicular. Foot Ankle Clin. 2010;15(2):337–47. doi: 10.1016/j.fcl.2010.02.004 20534360

[8] Nakayama S, Sugimoto K, Takakura Y, Tanaka Y, Kasanami R. Percutaneous drilling of symptomatic accessory navicular in young athletes. Am J Sports Med. 2005;33(4):531–5. doi: 10.1177/0363546504270564 15722276

[9] Candan B, Torun E, Dikici R. The Prevalence of Accessory Ossicles, Sesamoid Bones, and Biphalangism of the Foot and Ankle: A Radiographic Study. Foot Ankle Orthop. 2022;7(1):24730114211068792. doi: 10.1177/24730114211068792 35097490 PMC8777356

[10] Coskun N, Yuksel M, Cevener M, Arican RY, Ozdemir H, Bircan O, et al. Incidence of accessory ossicles and sesamoid bones in the feet: a radiographic study of the Turkish subjects. Surg Radiol Anat. 2009;31(1):19–24. doi: 10.1007/s00276-008-0383-9 18633564

[11] Miller TT. Painful accessory bones of the foot. Semin Musculoskelet Radiol. 2002;6(2):153–61. doi: 10.1055/s-2002-32361 12077704

[12] Huang J, Zhang Y, Ma X, Wang X, Zhang C, Chen L. Accessory navicular bone incidence in Chinese patients: a retrospective analysis of X-rays following trauma or progressive pain onset. Surg Radiol Anat. 2014;36(2):167–72. doi: 10.1007/s00276-013-1158-5 23897536

[13] Matsuishi T, Nagano M, Araki Y, Tanaka Y, Iwasaki M, Yamashita Y, et al. Scale properties of the Japanese version of the Strengths and Difficulties Questionnaire (SDQ): a study of infant and school children in community samples. Brain Dev. 2008;30(6):410–5. doi: 10.1016/j.braindev.2007.12.003 18226867

[14] Okuda M, Sasaki S, Bando N, Hashimoto M, Kunitsugu I, Sugiyama S, et al. Carotenoid, tocopherol, and fatty acid biomarkers and dietary intake estimated by using a brief self-administered diet history questionnaire for older Japanese children and adolescents. J Nutr Sci Vitaminol (Tokyo). 2009;55(3):231–41. doi: 10.3177/jnsv.55.231 19602831

[15] Liu L, Wang T, Qi H. Foot pain in children and adolescents: a problem-based approach in musculoskeletal ultrasonography. Ultrasonography. 2024;43(3):193–208. doi: 10.14366/usg.24002 38644779 PMC11079505

[16] Veitch JM. Evaluation of the Kidner procedure in treatment of symptomatic accessory tarsal scaphoid. Clin Orthop Relat Res. 1978(131):210–3. 657625

[17] Vavken P, Ganal-Antonio AK, Quidde J, Shen FH, Chapman JR, Samartzis D. Fundamentals of Clinical Outcomes Assessment for Spinal Disorders: Clinical Outcome Instruments and Applications. Global Spine J. 2015;5(4):329–38. doi: 10.1055/s-0034-1396046 26225283 PMC4516739

[18] Vavken P, Ganal-Antonio AK, Shen FH, Chapman JR, Samartzis D. Fundamentals of clinical outcomes assessment for spinal disorders: study designs, methodologies, and analyses. Global Spine J. 2015;5(2):156–64. doi: 10.1055/s-0035-1547525 25844291 PMC4369198

[19] Lee JH, Kyung MG, Cho YJ, Go TW, Lee DY. Prevalence of Accessory Bones and Tarsal Coalitions Based on Radiographic Findings in a Healthy, Asymptomatic Population. Clin Orthop Surg. 2020;12(2):245–51. doi: 10.4055/cios19123 32489548 PMC7237265

[20] Tsuruta T, Shiokawa Y, Kato A, Matsumoto T, Yamazoe Y, Oike T, et al. [Radiological study of the accessory skeletal elements in the foot and ankle (author’s transl)]. Nihon Seikeigeka Gakkai Zasshi. 1981;55(4):357–70. 7276670

[21] Alsager GA, Alzahrani K, Alshayhan F, Alotaibi RA, Murrad K, Arafah O. Prevalence and classification of accessory navicular bone: a medical record review. Ann Saudi Med. 2022;42(5):327–33. doi: 10.5144/0256-4947.2022.327 36252147 PMC9557784

[22] Bahl A, Bagan M, Joseph S, Brackney A. Comparison of Ultrasound and Plain Radiography for the Detection of Long-bone Fractures. J Emerg Trauma Shock. 2018;11(2):115–8. doi: 10.4103/JETS.JETS_82_17 29937641 PMC5994859

[23] Moritz JD, Berthold LD, Soenksen SF, Alzen GF. Ultrasound in diagnosis of fractures in children: unnecessary harassment or useful addition to X-ray? Ultraschall Med. 2008;29(3):267–74. doi: 10.1055/s-2008-1027329 18516770

[24] Endara-Mina J, Kumar H, Ghosh B, Mehta A, Chandra Dey R, Singh P, et al. Comparative use of ultrasound and radiography for the detection of fractures: a systematic review and narrative synthesis. Ann Med Surg (Lond). 2023;85(10):5085–95. doi: 10.1097/MS9.0000000000001229 37811018 PMC10553010

[25] Kalbouneh H, Alajoulin O, Alsalem M, Humoud N, Shawaqfeh J, Alkhoujah M, et al. Incidence and anatomical variations of accessory navicular bone in patients with foot pain: A retrospective radiographic analysis. Clin Anat. 2017;30(4):436–44. doi: 10.1002/ca.22876 28295608

[26] Sella EJ, Lawson JP, Ogden JA. The accessory navicular synchondrosis. Clin Orthop Relat Res. 1986(209):280–5. 3731610

[27] Choi YS, Lee KT, Kang HS, Kim EK. MR imaging findings of painful type II accessory navicular bone: correlation with surgical and pathologic studies. Korean J Radiol. 2004;5(4):274–9. doi: 10.3348/kjr.2004.5.4.274 15637478 PMC2698172

[28] Özbalcı AB, Erdoğan F, Coskun HS. Avascular Necrosis of the Type II Accessory Navicular Bone: A Rare Case Report. J Am Podiatr Med Assoc. 2022;112(3):21–040. doi: 10.7547/21-040 35797231

[29] Mosel LD, Kat E, Voyvodic F. Imaging of the symptomatic type II accessory navicular bone. Australas Radiol. 2004;48(2):267–71. doi: 10.1111/j.1440-1673.2004.01286.x 15230772

